# Immune cell profiling reveals diverse niches of immune residents of the enteric nervous system and potential neuroimmune interactions

**DOI:** 10.1073/pnas.2413692122

**Published:** 2025-06-23

**Authors:** Haozhe Wang, Aidil Zaini, Bailey Cardwell, Matthew C. Rowe, Alana Butler, Connie H. Y. Wong, Daniel P. Poole, Benjamin Marsland, Joel C. Bornstein, Nicola L. Harris

**Affiliations:** ^a^Department of Immunology, School of Translational Medicine, Monash University, Melbourne 3004, VIC, Australia; ^b^Drug Discovery Biology Theme, Monash Institute of Pharmaceutical Sciences, Monash University, Parkville 3052, VIC, Australia; ^c^Centre for Inflammatory Diseases, Department of Medicine, School of Clinical Sciences at Monash Health, Monash Medical Centre Monash University, Clayton 3168, VIC, Australia; ^d^Department of Anatomy and Physiology, University of Melbourne, Melbourne 3010, VIC, Australia

**Keywords:** neuroimmune interactions, gastrointestinal tract, enteric nervous system, GI homeostasis

## Abstract

Gastrointestinal (GI) neuroimmune interactions are crucial sensors and regulators of tissue homeostasis. Most enteric neurons reside within the myenteric plexus of the enteric nervous system in the muscular region, forming a structure called the *muscularis externa*. Despite established interactions between muscularis macrophages and neurons, the presence and function of other immune cell types remains poorly characterized. Here, we mapped the muscularis immune cell landscape, revealing that diverse cell types are present within distinct locations of the GI tract, and they lie in proximity to neuronal cell bodies and their axons. Using a hypothesis-free computational approach, we identify putative ligand–receptor interactions from publicly available single-cell RNA datasets and further validate one of these (App-CD74). This study provides a valuable reference to encourage new avenues of research underpinning enteric neuroimmune interactions as key contributors to GI homeostasis and diseases.

The enteric nervous system (ENS) is semiautonomous and regulates Gastrointestinal (GI) motility, fluid secretion, and nutrient absorption. Within the ENS, enteric neurons are anatomically separated into two interconnected plexuses—the myenteric and submucosal plexuses. Most enteric neurons reside in the myenteric plexus between the circular and longitudinal muscles, forming a structure known as the muscularis (1). Muscularis macrophages (MMs) interact bidirectionally with enteric neurons and are crucial for ENS functions (2). Recent single cell RNA sequencing (scRNAseq) profiling of enteric neurons (3, 4) and muscularis immune cells (5, 6) uncovered diverse neuronal and immune cell subsets beyond those previously characterized. To establish a comprehensive, data-driven framework for understanding region-specific GI neuroimmune interactions, we systematically profiled ileal and colonic muscularis immune cell subsets by flow cytometry and confirmed their proximity to neurons using immunohistochemistry, thereby spatially validating that direct interactions between muscularis neurons and niche-specific immune cells are possible. Using well-established bioinformatic approaches, we then used publicly available scRNAseq data from muscularis neurons and immune cells to predict biologically relevant ligand–receptor interactions within regionally distinct areas of the intestine. The relevance of one of the interactions (App-CD74) was further validated at a protein and spatial level. This study collectively provides a comprehensive reference of GI muscularis neuroimmune crosstalk during homeostasis.

## Results and Discussion

**Fig. 1.**
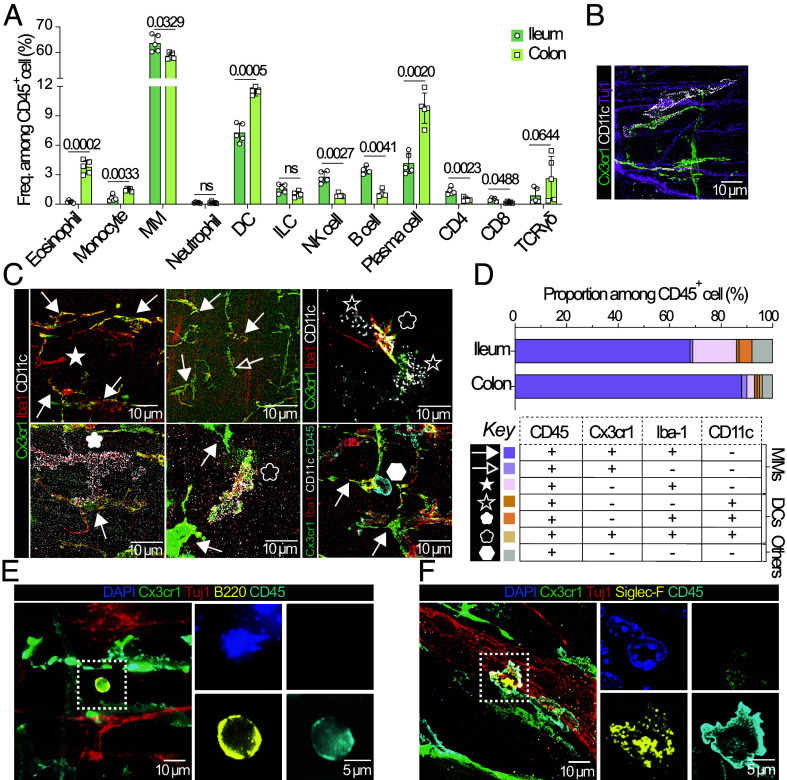
Immune cell landscape of healthy ileal and colonic muscularis. (*A*) Frequencies of indicated muscularis immune cell subsets among CD45^+^ cells. (*B*) Representative image of ileal and colonic muscularis indicating DCs (CD11c^+^) and MM (Cx3cr1^+^) contacting neuronal axons (Tuj1^+^). (*C*) Representative images of muscularis stained with indicated antibodies (symbols indicate cells of interest indicated in *D*). (*D*) Quantification of immune cell subsets identified in (*C*). Data generated by counting 150 to 360 CD45+ cells per image (>0.68 mm2) across 4 images. (*E*) Ileal muscularis stained with antibodies identifying B cells. (*F*) Colonic muscularis stained antibodies identifying eosinophils. Data from panel (*A*) are representative of three independent experiments (n = 4-5 mice per group). Statistical significance of data comparing ileum vs. colon was determined with a Paired *t* test. MM: muscularis macrophage; NK: natural killer; DC: dendritic cell; PC: plasma cell; ILC: innate lymphoid cell. All images were taken as Z-stacks of the whole muscularis and are presented as maximum intensity Z-projections.

Flow cytometry analysis of physically extracted and enzymatically digested muscularis revealed that macrophages were the dominant cell type residing in the muscularis of both the ileum and colon but that a rich diversity of other immune cells e.g., monocytes, dendritic cells (DCs), innate lymphoid cells (ILCs), eosinophils, plasma cells (PCs), natural killer (NK), T and B cells (Fig. 1*A*) were also present. In both the ileal and colonic muscularis, DCs and PCs formed the most abundant subsets after macrophages (Fig. 1*A*). Location influenced the immune cell repertoire with the colonic muscularis harboring higher proportions of eosinophils, gamma delta T cells, monocytes, DCs, and PCs, whereas the ileal muscularis contained higher frequencies of macrophages, B, NK, and T cells, especially CD4 T cells (Fig. 1*A*).

Next, we performed immunohistochemistry and confocal microscopy to determine whether the immune cells identified by flow cytometry were present in the myenteric plexus or muscle. Using the pan-leukocyte marker, CD45, we observed that most immune cells were located between the myenteric plexus (identified by the pan-neuronal marker, PGP9.5) and the circular muscle (Movies S1 and S2), with some CD45^+^ cells infiltrating myenteric ganglia (distinct clusters of nerve cell bodies, Movies S3 and S4). This indicates that close contact between immune cells and neuronal cell bodies is possible.

We used immunohistochemistry to provide an independent cross-validation of immune cell proportions identified by flow cytometry. Using expression of Cx3cr1 or CD11c as bona fide markers of CD45^+^ MMs and DCs and their bipolar or stellate morphologies, we showed these cells can contact Tuj1^+^ neuronal axons (Fig. 1*B*). Distinct MM and DC populations were further identified on the basis of Iba-1 expression (Fig. 1*C*). Cx3cr1^+^Iba-1^+^CD11c^−^ MMs were the most abundant population followed by Cx3cr1^+^Iba-1^−^CD11c^−^ MMs (Fig. 1*D*). CD11c^+^ DCs included both Iba-1^+^ and Iba-1^−^ populations and a rare population of Cx3cr1^+^Iba-1^+^ cells (7, 8) (Fig. 1*D*). In contrast to our flow cytometry findings, the immunohistochemistry analysis revealed a smaller fraction of non-MM immune cells (Fig. 1*A* vs. Fig. 1*D*). This discrepancy likely resulted from a selective loss of MMs (>70 µm) during the tissue disassociation and cell filtration processes required for flow cytometry, resulting in the underrepresentation of MM abundance (Fig. 1*A* vs. Fig. 1*D*). We also identified CD45^+^ Cx3cr1^−^ B220^+^ B cells and CD45^+^ Cx3cr1^−^ Siglec-F^+^ eosinophils with multilobed nuclei located adjacent to nerve fibers (Fig. 1 *E* and *F*). Notably, eosinophils were located exclusively within the terminal segment of the proximal colon and the beginning of mid colon (Fig. 1*F* and Movie S5), which are critical for water reabsorption and have unique microbial characteristics (9). This may reflect niche-specific neuroimmune interactions as eosinophils express neuromedin U (NMU) receptor 1 (10) that can interact with enteric cholinergic neurons expressing NMU.

Next, we determined potential neuroimmune interactions using CellChat (11) by integrating previously published scRNAseq datasets of small intestinal (Dataset S1) and colonic (Dataset S2) myenteric neurons (3, 4) with the respective muscularis immune cells (5, 6). We explored the ability of immune cells and neurons to act as senders (expressing ligands) or receivers (expressing receptors or coreceptors). Our analysis predicted that senders including DCs, T cells, neutrophils, and macrophages interact with all types of enteric neurons (Dataset S1). NK cells, ILCs, and PCs exhibited only a small number of interactions (Dataset S1). By contrast, we predicted higher interaction strengths when neurons act as senders, with all neuronal subsets expressing ligands able to signal to immune cells (Dataset S1). Although this could be attributed to technical limitations inherent to the depth of scRNAseq and that gene expressions may not always correlate with protein levels, it is plausible to hypothesize that neuroimmune dialogs are more likely to occur from the neuron to the immune cell than vice versa.

**Fig. 2.**
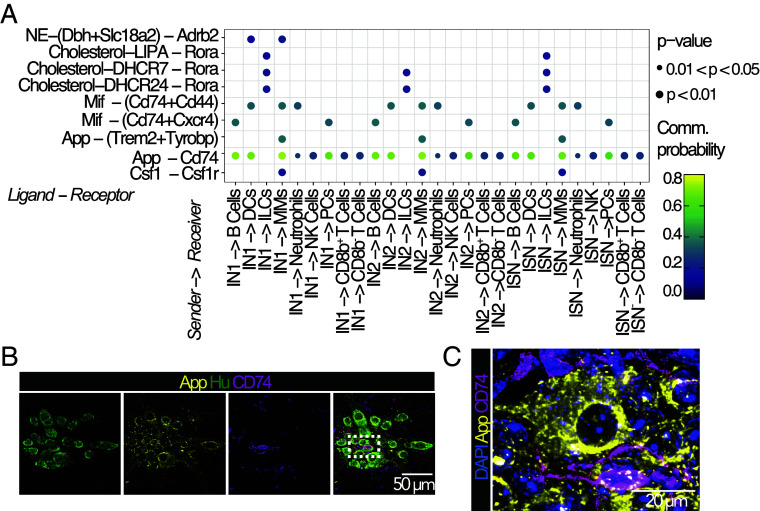
Neuroimmune interactions in the small intestine. (*A*) Specific ligand–receptor interactions predicted when the sender (ligand expressing) is a neuron, and the receiver is an immune cell. Node color represents communication probability; node size represents the p-value. IN1, IN2, and ISN neuronal subsets show similar App–CD74 interactions as EMN and IMN (Datasets S1 and S2). (*B*) Ileal muscularis stained with antibodies against App, CD74, neuronal cell bodies (Hu^+^), and DAPI, (*C*) indicating CD74^+^ cells contacting neurons (App^+^). MM: muscularis macrophages; NK: natural killer; DC: dendritic cell; PC: plasma cell; ILC: innate lymphoid cell; ISN: intrinsic sensory neurons; IN1: interneuron 1—cholinergic, nitrergic; IN2: interneuron 2—somatostatin; IMN: inhibitory motor neuron; EMN: excitatory motor neuron (3).

Network analysis further predicted specific ligand–receptor interactions (Datasets S1 and S2). Most ligand–receptor interactions were unique to distinct immune cell subsets when immune cells are considered as senders, except for DCs and macrophages that both expressed prosaposin (encoded by *Psap*) and granulin (encoded by *Grn*) (Dataset S1). Prosaposin and granulin promote neuronal branching, regeneration, and survival (12, 13), indicating that DCs and macrophages may be involved in these neuroprotective features. Similarly, ligand–receptor interactions were noted across distinct neuronal subsets when neurons act as senders (Fig. 2*A* and Datasets S1 and S2). For example, Csf1-Cfs1r signals were predicted between macrophages and neuronal subsets such as intrinsic sensory neurons (ISN) and interneurons (IN), which is consistent with a central role for this growth factor in the survival of MMs (2) (Fig. 2*A*). Despite the relatively low abundance of ILCs within the muscularis, cholesterol-mediated interactions were predicted from all neuronal subtypes uniquely to retinoic acid-related orphan receptor alpha (RORa)-expressing ILCs (Fig. 2*A*). This extends the already known ability of neurons to communicate with ILCs via neuropeptides such as NMU (1).

Unexpectedly, we predicted strong β-Amyloid precursor protein (encoded by *App*) from all neuronal subsets to interact with CD74, which is the invariant chain of class II major histocompatibility complex that can also act as an APP coreceptor (14) (Fig. 2*A*) in both ileum and colon (Datasets S1 and S2). We performed immunohistochemistry to validate APP and CD74 protein expression (Fig. 2*A*) and observed APP-expression on neurons in contact with CD74^+^ immune cells (Fig. 2*C*). Based on their stellate morphologies, it is likely that these immune cells are MMs or DCs (Fig. 2*C*). APP can regulate intestinal immune responses (15), and our data imply a possible role for CD74 in this process.

Collectively, our data map the immune landscape and predicts ligand–receptor interactions of the region-specific intestinal muscularis, implicating previously unappreciated neuroimmune crosstalk. Many of the immune cell subsets were found in proximity to neurons, and key neuroimmune interactions were predicted using bioinformatic approaches, with App and CD74 expressions spatially validated. Recognizing that mechanistic or functional validation lies beyond the scope of this study, our dataset establishes a data-driven framework for future functional or mechanistic studies on neuroimmune interactions and their roles during GI homeostasis or diseases.

## Materials and Methods

Eight- to ten-week-old male C57/BL6 and *Cx3cr1^gfp/+^* mice were used for muscularis immune profiling and immunohistochemistry. Physically extracted muscularis tissues were digested and acquired for flow cytometry or prepared for wholemount staining and immunofluorescence. Transcriptomic profiles of neurons and immune cells were integrated from published scRNAseq data and used for CellChat to predict ligand–receptor interactions. All animal studies were approved by the Alfred Research Alliance Animal Ethics Committee and Monash Medical Centre Animal Ethics Committee (Melbourne, Australia). Refer to detailed procedures and data processing, as described in *SI Appendix*.

## Supplementary Material

Appendix 01 (PDF)

Dataset S01 (XLSX)

Dataset S02 (XLSX)

Movie S1. Orthogonal view of the ileal muscularis. Tissue was stained with antibodies against the panneuronal marker PGP9.5 (red) and immune cell marker CD45 (green). A Z-stack image was resliced to generate the orthogonal view. CM: circular muscle; MP: myenteric plexus; LM: longitudinal muscle.

Movie S2. Orthogonal view of the colonic muscularis. Tissue was stained with antibodies against the pan-neuronal marker (PGP9.5, red) and immune cell marker (CD45, green). A Z-stack image was resliced to generate the orthogonal view. CM: circular muscle; MP: myenteric plexus; LM: longitudinal muscle.

Movie S3. 3D visualization of immune cell infiltration in the ileal ganglion. Tissue was stained with antibodies against the pan-neuronal marker (PGP9.5, red) and immune cell marker (CD45, green). The 3D view was reconstructed from a Z-stack image of the entire ileal muscularis.

Movie S4. 3D visualization of immune cells infiltration in the colonic ganglion. Tissue was stained with antibodies against the pan-neuronal marker (PGP9.5, red) and immune cell marker (CD45, green). The 3D view was reconstructed from a Z-stack image of the entire colonic muscularis.

Movie S5. The presence of eosinophils in the colonic muscularis. Different compartments of the colonic muscularis (from proximal to distal) were stained with antibodies against the eosinophil marker SiglecF (green) and the pan-neuronal marker Tuj1 (magenta). Z-stack images of each compartment were acquired across the full thickness of the muscularis and are presented as maximum intensity projections.

## Data Availability

All study data are included in the article and/or supporting information.
