# Supplementary material for: Immune cell profiling reveals diverse niches of immune residents of the enteric nervous system and potential neuroimmune interactions
Source: Proc Natl Acad Sci U S A. 2025 Jun 23;122(26):e2413692122. doi: 10.1073/pnas.2413692122 (PMC12232633; doi:10.1073/pnas.2413692122)
Supplement: Supplementary file 1 — Appendix 01 (PDF) [file pnas.2413692122.sapp.pdf]

PNAS

Supporting Information Appendix for

Immune cell profiling reveals diverse niche-specific immune residents of the enteric nervous system and potential neuroimmune interactions.

Haozhe Wang[a, 1], Aidil Zaini[a, 1], Bailey Cardwell[a], Matthew C. Rowe[b], Alana Butler[a], Connie H.Y. Wong[c], Daniel P. Poole[b], Benjamin Marsland[a], Joel C. Bornstein[d], Nicola Harris[a, 2]

[a] Department of Immunology, School of Translational Medicine, Monash University, Melbourne, Victoria, Australia

[b] Drug Discovery Biology, Monash Institute of Pharmaceutical Sciences, Monash University, Parkville, VIC 3052, Australia

[c] Centre for Inflammatory Diseases, Department of Medicine, School of Clinical Sciences at Monash Health, Monash Medical Centre Monash University Clayton Victoria Australia

[d] Department of Anatomy and Physiology, University of Melbourne, Melbourne, Victoria, Australia

[1] H.W and A.Z contributed equally to this work.

[2] Corresponding author: Nicola Harris

Email: nicola.harris@monash.edu

This file includes:

Material and Method
Reference

Supporting Information Appendix

Extended Methods

Animals and tissue collection

8 to 10 weeks old male C57/BL6 and Cx3cr1$^{gfp/+}$ mice were acquired from the Monash Animal Research Platform or bred in-house and housed in the Monash Intensive Care Unit (ICU) facilities or Monash Medical Centre Animal Facility within the specific pathogen-free environment. Briefly, mice were humanely killed by cervical dislocation, and intestines were collected without damaging the serosa. The ileum was defined as the last 5 to 7 cm of the small intestine (i.e., measured from the distal end). The colon was collected after removing the first 1 cm (cecal-colon junction) and the last 1 cm (rectum) of the tissue. Excess mesenteric fat was removed, and tissues were cut open along the mesenteric border and kept in ice-cold PBS for further processing. All experiments were approved by the Alfred Research Alliance Animal Ethics Committee (Melbourne, Australia).

Tissue digestion and flow cytometry

Tissues were stretched and pinned to a Sylgard petri-dish filled with phosphate-buffered saline with the mucosal side facing down. The *muscularis externa* was collected by gently peeling using fine forceps under a dissection microscope. The tissue was chopped into small pieces and digested with HBSS containing Mg^{2+}/Ca^{2+} with 5% FCS, 2 mg/ml Collagenase IV, 2 mg/ml Dispase, 25 mM HEPES, 1 mM NaPyr, 50 U/mL DNase I at 37°C for 40 min on a shaker. Tissues were then homogenized with an 18G needle/10 mL syringe by aspirating up and down twice, then filtered through a 70 µm cell strainer to form single-cell suspensions. Of note, the use of a 70 µm filter might have excluded larger or structurally larger cells with dendrite-like structures. Therefore, it is likely that certain large macrophages—such as those exceeding 70 µm due to their dendriform processes—might not pass through the filter or might be compromised during tissue dissociation. Tissues were stained using the following antibody panel: CD45-BUV395 (1:400), CD11b-PerCP-Cy5.5 (1:400), CD11c-PE-Cy7 (1:400), F480-BUV737 (1:500), Siglec F-PE-C594 (1:200), Ly6C-BV510 (1:200), Ly6G-BV711 (1:200), CD4-PE (1:300), CD8b-FITC (1:200), FCR1a-AlexaFluor700 (1:200), TCRγδ-BUV563 (1:200), TCRβ-APC (1:200), MHCII-APC Cy7 (1:200), CD98-Alexa647 (1:200), CD19-BV421 (1:200), NK1.1-PE-Cy5 (1:200), CD127-BV786 (1:200), CD90.2-BV605 (1:200), CD206-BV650 (1:200). Data was acquired on the Cytek Aurora CS and analyzed using FlowJo (BD Biosciences).

Immunohistochemical staining, image acquisition, and image analysis

Tissues were stretched and pinned with the mucosal side facing up and fixed with 4% PFA overnight. The *muscularis externa* was collected by peeling away the mucosa and submucosa under a dissection microscope. Tissues were incubated with a blocking buffer (5% donkey serum, 0.5% bovine serum albumin, 0.3% Triton X-100, 0.1% NaN_3 in PBS) for 30 min. Tissues were then incubated overnight with primary antibody mixed in blocking buffer, and then followed by an overnight secondary incubation in blocking buffer. All staining incubations were performed on a shaker at 4°C. The primary antibodies used for immunohistochemical staining were: rat anti B220-PE (1:100, BioLegend, #103208), Armenian hamster anti CD11c-APC (1:100, BioLegend, #117310), Armenian hamster anti CD11c-PE (1:100, BioLegend, #117308), goat anti CD45 (1:100, R&D Systems, #AF114), Rat anti F4/80 (1:100, Abcam, #ab6640), mouse anti PGP9.5 (1:100, Abcam, #ab8189), rabbit anti Tuj1 (1:100, Abcam, #ab18207), rat anti SiglecF (1:100, BD biosciences, #552125), rabbit anti GFP(1:400, Thermo Fisher A-21311), rabbit anti APP (1: 500, Abcam, #ab32136), rat anti CD74 (1:500, BioLegend, #151002), rabbit anti Iba-1 (1:500, Fujifilm Wako, #019-19741), human anti Hu (ANNA1) (1:5,000 Gift from Dr. V. Lennon). The secondary antibodies used for immunohistochemical staining were: donkey anti mouse AF488 (1:200, Jackson ImmunoResearch, #705-545-147), donkey anti mouse AF568 (1:200, Thermo Scientific, #A10037), donkey anti rat AF488 (1:500, Thermo Scientific, #A10042), donkey anti rat AF647 (1:500, Jackson ImmunoResearch, #712-605-153), donkey anti rabbit AF568 (1:500, Thermo Scientific, #A21447), donkey anti rabbit AF647 (1:500, Jackson ImmunoResearch, #711-605-152), donkey anti rabbit AF750 (1:250, Abcam, #ab175728), donkey anti goat AF488 (1:500, Jackson ImmunoResearch, #705-545-147), donkey anti goat (AF647, 1:500, Thermo Scientific, #A21208), donkey anti goat AF750 (1:250, Abcam, #ab175745), donkey anti human AF488 (1:500, Jackson ImmunoResearch, #709-545-149), donkey anti human Rhodamine Red-X (1:500, Jackson ImmunoResearch, #709-295-149). To perform staining using two primary antibodies raised in the same species (species X), tissue was stained with the first X antibody following the protocol mentioned above. The tissue was then blocked with serum from species X for 4 h on a shaker at room temperature, followed by washing and then

incubation in donkey anti X fragment antibodies for 48 h. After washing, the tissue was then stained with the second X antibody as per the protocol outlined above.

Samples were mounted in DAKO mounting medium (Agilent, S3023) and imaged using a Nikon A1r confocal microscope or Leica DMi8 widefield microscope as Z-stacks (0.8 – 1.2 µm steps) using 20X or 40X objectives to cover the entire tissue depth. Images were analyzed using FIJI (ImageJ). For quantification, cells expressing CD45 were counted. Among these, immune cells that express CD11c, Iba-1, and/or Cx3cr1 were quantified and represented as a proportion of the total $CD45^+$ cells. For each tissue (ileum and colon), four images (> 0.65 mm² in area) were analyzed, and the proportions were calculated as the average of the four images. Orthogonal views of images were shown by re-slicing from the left of each image and saved as .avi format. 3D views of ileal and colonic ganglia were generated using the 3D viewer functions and saved as .avi format. Acquired widefield images were deconvolved using Huygens Professional Deconvolution Software (Version 24.04). Point spread functions (PSFs) were measured for each acquisition wavelength and magnification used on the Leica DMi8 widefield microscope. Measured PSFs were used to optimize the deconvolution process. All images were shown as a Z-project by maximum intensity projection.

Cell-Cell ligand-receptor pair inference

Transcriptomic profiles for each cell type were systematically compiled by reanalyzing published single-cell RNA (scRNA) sequencing data from the small intestine and colon myenteric neurons (1, 2) (GSE149524 and GSE156905) and muscularis externa $CD45^+$ immune cells (3, 4) (GSE167465 and GSE237862). All scRNAseq datasets included in this study utilized the 10x Genomics protocol (V2 or V3 – see original publications). Subsequently, we performed quality control procedures and analytical assessments within R (https://www.r-project.org/, version 4.3.1) using the Seurat R package (version 5.1.0)(15).

For all datasets, pre-processing was conducted on a per-study basis. Putative cells were filtered to remove low quality cells and doublets by inspection of the number of expressed features, number of UMI counts, and proportion of reads mapping to mitochondrial genes. Due to large differences in libraries between datasets, we manually chose filtering thresholds on a per-study basis. Then, doublets were removed following standard protocols as recommended by the authors of the 'DoubletFinder' (v2.0.4) package using data that had been normalized and processed with the scTransform() function of Seurat.

All datasets underwent standard Louvain clustering and were annotated using canonical markers of cell types identified in each respective study. All clusters were inspected for co-expression of contradictory cell markers and such spurious cells were removed. In some cases, cell types were collapsed or refined further from their original publications.

For the small intestine myenteric neuronal populations (GSE149524), we consolidated cells with analogous gene expression into cohesive clusters. This included excitatory MN (including ENC1, ENC2, ENC3 and ENC4; MN, motor neurons), inhibitory MN (including ENC8 and ENC9), ISN (including ENC6, ENC7 and ENC12; ISN, intrinsic sensory neuron), IN1 (ENC5, IN, interneuron), and IN2 (ENC10) (1). In the immune dataset (GSE167465) (3), cell annotations were manually curated based on gene markers as delineated from the original publication with further refinement of the T cell populations into T-cell and gamma-delta T-cells based on receptor chain expressions, and the B-cell populations into B-cell and plasma cells, to align with our flow cytometry data. For annotation, we included immune cells from both homeostatic and inflammatory states, due to the low cell number under the homeostatic state. For subsequent analysis, only gene expression profiles from healthy immune cells were utilized. It is worth noting that the number of cells obtained from scRNAseq analysis influences the downstream analysis of receptor-ligand interactions, as the probability of an interaction is dependent on the number of cells in each cluster.

Annotation of neuron (GSE156905) and immune (GSE237862) scRNAseq datasets of the mouse colon (2, 4) was performed in a similar manner to the small intestine dataset. We consolidated colonic myenteric neuronal populations into excitatory (Chat 1-3), inhibitory (Nos 1, Nos 2), intrinsic sensory neuron (Calcb), and interneurons (Chat 4), based on clustering from the original publication. For immune populations, clustering was performed on DSS and naive control samples and annotated based on expression of marker genes identified in the original publication. Only naive control samples were

used for subsequent CellChat analysis. This resulted in colonic immune populations of ILCs, DCs, macrophages, B cells, conventional T cells, and γδ T cells.

To identify potential ligand-receptor interactions between muscularis externa immune cells and myenteric neurons, we employed the R package CellChat (V2.1.2) to identify encoded and non-encoded mechanisms of cell-cell communication (5, 6). Transcriptional profiles of each annotated cell type were merged, and the resulting matrix underwent log normalization. CellChat was utilized using the default mouse database and default settings for all steps as described in the author's online vignettes. The 'triMean' method of communication probability calculations were used to retain higher confidence ligand-receptor pair interactions. Data were presented as dot plots showing computed interaction weight that indicates the strength of interactions derived from significant communication probabilities between each type of neuron and immune cells. CellChat statistics for all ligand-receptor pairs between neuron and immune populations for the small intestine and colon are presented in Datasets 1&2. As we did not find any neutrophils by flow cytometry, we removed neutrophils in the visualization of the receptor-ligand interactions, as indicated in dot plots. However, the data for neutrophils remains included in Dataset 1.

Statistical analyses

The differences between the ileum and the colon were analyzed with GraphPad Prism 10.2 using unpaired student-T tests. Significance levels of $p < 0.05$ (*), $p<0.01$ (**), $p<0.001$ (***) and $p<0.0001$ (****) were chosen. Error bars were shown as mean ± standard deviations.

Supplementary videos

SI Video 1. Orthogonal view of the ileal muscularis. Tissue was stained with antibodies against the pan-neuronal marker PGP9.5 (red) and immune cell marker CD45 (green). A Z-stack image was resliced to generate the orthogonal view. CM: circular muscle; MP: myenteric plexus; LM: longitudinal muscle.

SI Video 2. Orthogonal view of the colonic muscularis. Tissue was stained with antibodies against the pan-neuronal marker (PGP9.5, red) and immune cell marker (CD45, green). A Z-stack image was resliced to generate the orthogonal view. CM: circular muscle; MP: myenteric plexus; LM: longitudinal muscle.

SI Video 3. 3D visualization of immune cell infiltration in the ileal ganglion. Tissue was stained with antibodies against the pan-neuronal marker (PGP9.5, red) and immune cell marker (CD45, green). The 3D view was reconstructed from a Z-stack image of the entire ileal muscularis.

SI Video 4. 3D visualization of immune cells infiltration in the colonic ganglion. Tissue was stained with antibodies against the pan-neuronal marker (PGP9.5, red) and immune cell marker (CD45, green). The 3D view was reconstructed from a Z-stack image of the entire colonic muscularis.

SI Video 5. The presence of eosinophils in the colonic muscularis. Different compartments of the colonic muscularis (from proximal to distal) were stained with antibodies against the eosinophil marker SiglecF (green) and the pan-neuronal marker Tuj1 (magenta). Z-stack images of each compartment were acquired across the full thickness of the muscularis and are presented as maximum intensity projections.

References

1. K. Morarach, *et al.*, Diversification of molecularly defined myenteric neuron classes revealed by single-cell RNA sequencing. *Nat. Neurosci.* **24**, 34–46 (2021).

2. E. Drokhlyansky, *et al.*, The Human and Mouse Enteric Nervous System at Single-Cell Resolution. *Cell* **182**, 1606-1622.e23 (2020).

3. M. Stakenborg, *et al.*, Enteric glial cells favor accumulation of anti-inflammatory macrophages during the resolution of muscularis inflammation. *Mucosal Immunol.* **15**, 1296–1308 (2022).

4. K. Ohishi, *et al.*, Resolving resident colonic muscularis macrophage diversity and plasticity during colitis. *Inflamm. Bowel Dis.* (2024). https://doi.org/10.1093/ibd/izae155.

5. S. Jin, *et al.*, Inference and analysis of cell-cell communication using CellChat. *Nat. Commun.* **12**, 1088 (2021).

6. S. Jin, M. V. Plikus, Q. Nie, CellChat for systematic analysis of cell-cell communication from single-cell transcriptomics. *Nat. Protoc.* **20**, 180–219 (2025).
